# Celiac Disease: The Importance of Studying the Duodenal Mucosa-Associated Microbiota

**DOI:** 10.3390/nu16111649

**Published:** 2024-05-27

**Authors:** Alessandro Annunziato, Mirco Vacca, Fernanda Cristofori, Vanessa Nadia Dargenio, Giuseppe Celano, Ruggiero Francavilla, Maria De Angelis

**Affiliations:** 1 Department of Soil, Plant and Food Sciences, University of Bari Aldo Moro, Via Amendola 165/a, 70126 Bari, Italy; alessandro.annunziato@uniba.it (A.A.); giuseppe.celano@uniba.it (G.C.); maria.deangelis@uniba.it (M.D.A.); 2 Interdisciplinary Department of Medicine, Pediatric Section, Children’s Hospital ‘Giovanni XXIII’, University of Bari Aldo Moro, 70126 Bari, Italy; fernandacristofori@gmail.com (F.C.); vanessa.dargenio@unifg.it (V.N.D.); ruggiero.francavilla@uniba.it (R.F.)

**Keywords:** celiac disease, microbiome, mucosa-associated microbiota, gut, diet, gluten-free diet, dysbiosis

## Abstract

There is increasing evidence indicating that changes in both the composition and functionality of the intestinal microbiome are closely associated with the development of several chronic inflammatory diseases, with celiac disease (CeD) being particularly noteworthy. Thanks to the advent of culture-independent methodologies, the ability to identify and quantify the diverse microbial communities residing within the human body has been significantly improved. However, in the context of CeD, a notable challenge lies in characterizing the specific microbiota present on the mucosal surfaces of the intestine, rather than relying solely on fecal samples, which may not fully represent the relevant microbial populations. Currently, our comprehension of the composition and functional importance of mucosa-associated microbiota (MAM) in CeD remains an ongoing field of research because the limited number of available studies have reported few and sometimes contradictory results. MAM plays a crucial role in the development and progression of CeD, potentially acting as both a trigger and modulator of the immune response within the intestinal mucosa, given its proximity to the epithelial cells and direct interaction. According to this background, this review aims to consolidate the existing literature specifically focused on MAM in CeD. By elucidating the complex interplay between the host immune system and the gut microbiota, we aim to pave the way for new interventions based on novel therapeutic targets and diagnostic biomarkers for MAM in CeD.

## 1. Celiac Disease: Insights from Host Genetics to Gut Microbiota Implications

Celiac disease (CeD) is a chronic autoimmune condition triggered by gluten consumption leading to intestinal damage and systemic symptoms. The clinical manifestation of CeD includes both gastrointestinal symptoms (such as diarrhea, bloating, swelling, and abdominal pain) and extra-intestinal symptoms (including anemia, dermatitis herpetiformis, osteopenia, and peripheral neuropathy). As supported by studies on twins [1,2], although 95% of CeD patients possess HLA-DQ2 and HLA-DQ8 genes, which are pivotally involved in CeD development, their presence alone has been noted to be insufficient to ensure the disease development. As additional features of CeD, histological abnormalities (e.g., infiltration of inflammatory cells, villus atrophy, and crypt hyperplasia [3]) are assessed through endoscopic sampling.

Although data analysis suggests the potential for under-diagnosis leading to fluctuations in values of global incidence [4], the acknowledged prevalence of CeD ranges between 0.5 and 1% in Europe and North America, with a higher rate (2–3%) observed in Finland and Sweden [5]. On average, CeD is believed as a pediatric condition, with a peak incidence in children aged two to five years [6,7]. However, authors who attempted to validate this idea through specifically designed trials concluded that this hypothesis lacks solid foundations [7,8,9]. In fact, CeD can develop at any age, as well as in geriatric individuals [10], and some factors, including antibiotic use [11] and gastrointestinal viral infections [12] in early life, seem to be implicated in supporting its development.

Whatever the age of the clinical manifestation of CeD, the phenomenon known as “leaky gut” is thought to trigger early stages of innate immune activation allowing excessive trafficking of gluten-derived epitopes from the intestinal lumen to the lamina propria [13] and leading to an inflammation status as the result of the interaction between gliadin fragments and the intestinal lamina propria [14]. Subsequently, a cascade of immune responses occurs, characterized by the release of proinflammatory cytokines [15,16], particularly IL-15 [17].

In depicting the events occurring at the gut level, it is essential to mention an additional contributor: the gut microbiota (GM), which is a heterogeneous polymicrobial community, predominantly accounting for bacterial cells, as well as Archaea, eukaryotes (both yeasts and molds), protists, and viruses. In its totality, this so-called “superorganism” can encode various metabolic pathways that generate thousands of metabolites covering a broad spectrum of biological roles in hosts [18]. The composition of GM is based on cross-feeding mechanisms and additional factors such as pH, bile salts and other enzyme secretions, antibiotics, bacteriocins, and the substrate availability in the lumen. Therefore, each individual exhibits a unique GM fingerprint, which is driven and shaped during early life by different factors [19,20] that significantly impact during the first 1000 days of life [21].

Extensive research has shown the pivotal role of the GM in influencing the host’s overall well-being. From birth, the symbiotic relationship that occurs between the host and GM is crucial in programming the maturation of the immune system. Thereafter, GM plays a daily role in stimulating the optimal function of macrophages, dendritic cells, and neutrophils [22,23]. Additionally, GM is involved in synthesizing specific micronutrients, such as vitamins (K and B12), essential for the host and that cannot be obtained through dietary components alone. Furthermore, through its metabolism, the GM produces various molecules necessary for maintaining the integrity of the mucosal barrier and protecting against pathogens. In fact, extensive research on GM has highlighted how changes in its structure and functions are linked to numerous chronic inflammatory diseases [24], suggesting a potential relevance to CeD as well.

## 2. From Homeostasis to Dysbiosis: Gut Microbiota Implications in Health and Disease

The GM homeostasis is crucial for maintaining the resilience of the microbial community. This equilibrium is essential for overall health because GM can influence the development of both intestinal and systemic autoimmune diseases [25]. The maintenance of this balance relies on complex bidirectional interactions with the host.

Examined from a microbial ecology perspective, a healthy GM should demonstrate resilience against stressors and disturbances, and it should possess the ability to rebound to a healthy functional status [26,27,28]. In line with this, factors promoting GM resilience have the potential to improve human health [29,30]. Drafting a stable bacterial core GM, Shetty et al. [31] suggested that it should account for the presence of various genera such as *Bacteroides*, *Faecalibacterium*, *Eubacterium*, *Ruminococcus*, *Alistipes*, *Roseburia*, *Clostridium*, and *Blautia*. Additionally, specific species, such as *Faecalibacterium prausnitzii*, *Ruminococcus obeum*, and *Oscillospira guillermondii*, have been consistently found among healthy adults in different studies [31].

What is known is that when GM homeostasis is disrupted, it can lead to dysbiosis, a condition characterized by an imbalance in the microbial community featured by loss in commensal and keystone taxa with a bloom of pathobionts [32,33]. From this understanding, dysbiosis has been linked to various health issues [24]. However, it is important to note that dysbiosis cannot simply be defined as the presence, absence, or abundance of specific taxa because it is intricately related to the host’s pathophysiological condition. As an example, in individuals suffering from metabolic disorders, whatever microorganism is able to uptake excessive energy from dietary components can be potentially dangerous for the host’s health [34] while, in nephropathic patients, whatever microorganism possessing the metabolic pathway/s for utilizing ammonia to metabolize uremic toxins can act as a contributor for the kidney failure progression [35]. Therefore, in the case of CeD, an increase in proteolytic microorganisms can exacerbate the immune response by hydrolyzing gluten into immunotoxic peptides [36].

## 3. Why to Study the Duodenal Mucosa-Associated Microbiota (MAM)

The nutrient availability within the mucus layer of the epithelium significantly differs from that in the gut lumen, leading to notable differences in diversity and composition between the mucosa-associated microbiota (MAM) and microbes residing in the intestinal lumen, delineating two distinct microbial niches [37]. Supported by earlier studies involving HCs [38], this observation supports the belief that MAM plays a crucial role in stimulating the host immune system [39]. Because MAM resides proximally to the intestinal epithelium, it is likely to engage in more direct interactions with the host immune system than luminal or fecal microbes [40]. Therefore, in the context of CeD, where the immune system’s pivotal role in the disease has been established, bidirectional interactions with MAM are plausible. Moreover, previous studies have suggested that MAM profiling may yield more consistent results than fecal samples for comprehensive profiling [41]. However, limited studies of MAM in humans have been conducted due to methodological challenges, most notably, the need for Ethical Committee validation for diseases where endoscopic methodologies are unnecessary for diagnosis or treatment.

## 4. Unravelling the Microbiota Fingerprint in CeD under a Gluten-Containing Diet

CeD is a complex condition based on a multifaced etiology among which the role of gluten is pivotal despite its influence during the CeD development is still not completely understood.

Herein, we reviewed the specific MAM fingerprint featuring the proximal tracts of the intestine of CeD patients under a gluten-containing diet (GCD). The narrative dissertation based on a systematic article selection was included in Section 4.3.1 and Section 4.3.2. The workflow used to screen and select ideal research papers is detailed in Appendix A and Supplementary Figure S1. Noteworthy, during the systematic selection, all results and studies involving CeD patients adhering to gluten-free diet (GFD) were excluded to avoid bias associated with MAM composition influenced by this specific dietary regimen. Although not included in the systematic article selection, to complete the overview concerning the GM in CeD, evidence concerning both the oral and fecal microbiota was discussed in Section 4.1 and Section 4.2, respectively.

### 4.1. Oral Microbiota in CeD

The oral cavity serves as both the initial digestive organ for incoming food and the second environment where microorganisms thrive. More than 1000 different bacteria inhabit the mouth, finding residence in our teeth, gums, and saliva [3]. Recent studies propose that these “friendly mouth microbes” fortify the mouth’s lining, bolstering its defenses against harmful invaders [42]. Notably, certain genera such as *Veillonella* and *Streptococcus* appear pivotal in generating antimicrobial peptides and inflammatory signaling molecules [42]. However, the narrative extends further. Investigations, such as that conducted by Atarashi et al. [43], suggest that specific oral microbes, such as *Klebsiella*, can migrate to the gut, potentially eliciting inflammatory responses by modulating immune cells. Recently, Panelli et al. [44] discussed a significant overgrowth of Proteobacteria in active CeD, with *Neisseria* and *Acinetobacter* spp. identified as the most representative taxa in salivary samples. Noteworthy, the authors pointed out how the bacterial microbiota in the saliva better aligned with the duodenal mucosa microbiota, rather than with fecal samples [44].

Interestingly, research also indicates a plausible relationship between oral bacteria and CeD [45]. Salivary microbiota may modulate CeD risk by modifying gliadin peptides through enzymatic activity since oral bacteria capable of degrading gluten have been identified in dental plaque and saliva, suggesting a role in CeD pathogenesis [46]. This supports the idea that proteolytic activities of bacteria, including those in the oropharyngeal tract, influence individual immunoreactivity to gluten. A higher gluten substrate hydrolysis in the saliva of CeD patients implies that oral microbe-derived enzymes can affect gluten peptide processing and antigen presentation to the intestinal immune system. Certain genera, including *Rothia*, *Actinomyces*, *Neisseria*, and *Streptococcus*, seem involved in the initial breakdown of gliadin, a component of gluten. Tian et al. recently found a bloom in Actinobacteria associated with a decrease in Bacteroidetes and Fusobacteria in the oral microbiome of individuals with refractory CeD [47]. Also, Iaffaldano et al. discussed microbial alterations in the throat and gut of CeD patients, characterized by a higher abundance of the Proteobacteria phylum and, at a lower taxonomic level, *Neisseria* spp. [48]. Additional studies investigated the oral/salivary microbiota in CeD [49,50,51]; however, since these based their research on CeD patients under GFD, we here not discuss their results to avoid bias-related microbial unbalances affected by the gluten-deprived dietary regimen more than that strictly related to the disease.

While some studies propose that HCs exhibit a higher rate of gluten degradation, particularly of the highly reactive 33-mer alpha-gliadin peptide, compared to CeD patients, the depiction is intricate. Oral microbial enzymes may degrade certain gluten proteins into smaller, potentially more immunogenic fragments, potentially exacerbating gut inflammation [47]. Mice genetically predisposed to CeD were more susceptible to disease development when gluten exposure was associated with certain bacterial enzymes (*Pseudomonas aeruginosa* elastases) [52], evidence that once more underscored the intriguing and multifaceted role of oral microbes in digestion and diseases, particularly in CeD.

### 4.2. Fecal Microbiota in CeD

In the last two decades, investigations of fecal samples from individuals with CeD utilized both culture-dependent and culture-independent methods [53]. The findings revealed a higher prevalence of *Bacteroides*/*Prevotella* cluster, *Clostridium histolitycum*, and *Eubacterium rectale*/*C. coccoides* group, and *Atopobium* genus in CeD patients [54]. Furthermore, specific species such as *Lactobacillus curvatus*, *Leuconostoc mesenteroides*, and *Leuconostoc carnosum* were identified in CeD patients [54], underscoring the necessity for further exploration of their fecal microbiota and proposing potential interventions with probiotics and prebiotics [18]. These results were corroborated by real-time PCR analysis, which illustrated a decrease in *Bifidobacterium* in CeD patients compared to HCs, alongside a higher abundance of specific bacterial groups, i.e., *Bacteroides* and *Clostridium leptum* (clostridial cluster IV), in both stool and biopsies of CeD patients [55]. Conversely, levels of *E. coli* and *Staphylococcus* were high in CeD patients consuming a gluten-containing diet (GCD) but normalized upon transitioning to a GFD. De Palma et al. [56] investigated the connection between gut microbiota and mucosal surface immunoglobulin secretion (IgA, IgG, IgM antibodies) in CeD patients, noting higher levels of *Bacteroides*/*Prevotella* taxa in CeD patients along with reduced IgA-coated bacteria, indicating compromised mucosal barrier function and heightened susceptibility to harmful antigens and pathogens. Further studies, including Nistal et al. [57], confirmed an incomplete restoration of GM after GFD, characterized by reduced diversity in *Lactobacillus* and *Bifidobacterium* species, as well as reduced concentrations of SCFAs compared to HCs, reflecting alterations in microbial composition. Additionally, SCFA metabolism only partially recovered after GFD, consistent with metabolomics studies in pediatric CeD patients [58]. Dysbiosis, linked to the reduced presence of protective bacteria, facilitates the proliferation of opportunistic pathogens carrying virulent genes, such as *S. epidermidis*, which harbors the mecA gene, observed in both GCD and GFD patients compared to controls [59].

### 4.3. MAM

To deeply inspect differences in MAM taxonomical composition, we conducted a literature search based on the use of specific keywords combined with Boolean operators, as detailed below (Appendix A). After filtering and manual check, all pertinent original articles were studied and elaborated in Section 4.3.1 and Section 4.3.2 with the aim to describe the MAM dysbiosis in CeD patients on GCD.

#### 4.3.1. MAM in Pediatric CeD Patients

Since 2007, studies have consistently documented MAM in children with CeD compared to HCs (Table 1). The first study, carried out by Nadal et al. [60], reported a significantly higher total bacterial count, mainly accounting for Gram-negative, in active CeD patients than HCs. Also, authors noticed a significative detection of *Bacteroides*/*Prevotella* spp. and *E. coli* in active CeD children [60]. Schippa et al. corroborated these results based on *Bacteroides* spp. and *E. coli* which were significantly more abundant in biopsy specimens of CeD children [61]. Fernandez-Crehuet et al. [62] showed that the band-based profile of CeD mainly accounted for the detection of *Streptococcus*, *Bacteroides*, and *E. coli*, whereas HCs for *Bifidobacterium*, *Acinetobacter*, and *Lactobacillus.*

Due to the absence of significance in MAM composition between CeD children and HCs in the study carried out by Cheng et al. [63], authors identified a possible health status-related bacterial sub-population by running a Random Forests analysis. Through this approach, the study suggested how higher values of specific features (*Prevotella melaninogenica* et related, *Haemophilus* ssp., and *Serratia* ssp.) can provide a way to differentiate active CeD from HCs. Similarly, although not significant, Nistal et al. observed an increase in *Neisseria* and *Haemophilus* with reduced *Streptococcus* and *Prevotella* detection in CeD [57]. These findings almost aligned with those reported by Sanchez et al. [64], which found that microbes of the Proteobacteria phylum were more prevalent in CeD patients HCs, whereas Firmicutes showed the opposite. Deeply, certain families, such as *Enterobacteriaceae* (*Klebsiella* spp.) and *Staphylococcaceae* (*Staph. epidermidis* and *Staph. pasteuri*), were more abundant in CeD [64].

Based on RT-qPCR, Collado et al. reported no differences according to the prevalence of specific bacterial groups in biopsies from CeD compared to HCs [55]. However, the same study noticed a significantly lower abundance of *Bifidobacterium* and a higher abundance of both *Lachnospiraceae* and *Ruminococcaceae* (*Cl. coccoides* and *Cl. leptum* groups; i.e., clostridial cluster XIVa and IV, respectively), *Bacteroides*, *Staphylococcus*, lactobacilli and *E. coli* in CeD compared to paired-age HCs [55].

Analyzing MAM from the 2^nd^ part of the duodenum (D2), El Mouzan et al. found a large spectrum of significantly altered (log2 fold change) [65]. Compared to HC, *Flavobacteriaceae*, *Clostridiaceae*, and *Lactobacillaceae* were lower in CeD at the family level, whereas *Micrococcaceae* were higher. At the genus level, *Clostridium* was reduced in CeD, whereas *Lactobacillus*, *Subdoligranulum*, and *Kocuria* expanded [65]. Since the study was based on shotgun metagenomics, data at the species level were also analyzed statistically, revealing a depletion of *Roseburia intestinalis* in CeD. By contrast, *Bifidobacterium angulatum*, *Lactobacillus acidophilus*, *Acinetobacter lwoffii*, *Kocuria rhizophila*, *Ralstonia pickettii*, *Corynebacterium ihumii*, and *Corynebacterium tuberculostearicum* expanded significantly in CeD.

However, other studies did not corroborate the findings mentioned above, as their results did not achieve statistical significance. For instance, Kalliomäki et al. [66] observed no significant differences in the bacterial gene copies of various groups (including all Bacteria, the *Bifidobacterium* genus, the *Bacteroides-Prevotella-Porphyromonas* group, the *B. fragilis* group, the *Streptococcus* group, and the *Lactobacillus* group) and species (four species belonging to the *Bifidobacterium* genus and *Staphylococcus aureus*) between children with CeD and HCs. This finding is consistent with the data reported by Ou et al. [67] and De Meij et al. [68].

Noteworthy, Di Biase et al. also investigated the microbial alterations in the MAM associated with CeD children [69]. However, due to the absence of biopsy control samples for comparison, authors discussed only a dominant presence of microbes taxonomically belonging to the *Enterobacteriaceae* family in MAM samples from CeD without providing a value of significance. As a general description of the results, they also showed a decrease in keystone taxa in CeD, such as *Bacteroides* and *Streptococcus* but, once again, this conclusion was not derived by statistical comparison [69].

#### 4.3.2. MAM in Adult CeD Patients

The systematic article search and selection sheds light on studies that have examined MAM profiles in adults with CeD and compared them to those of HCs, as summarized in Table 2.

The studies conducted by Garcia-Mazcorro et al. [70] and Iaffaldano et al. [48] yielded similar findings at the phylum level, indicating a significant decrease in Bacteroidetes. Iaffaldano et al. [48] further observed that this decrease was mainly attributed to a lower relative abundance of *Prevotellaceae* at the family level, and *Prevotella* at the genus level. Both studies also suggested the involvement of Fusobacteria in describing the MAM in adult CeD. Garcia-Mazcorro et al. [70] reported a decreasing trend close to significance (*p* = 0.052), while Iaffaldano et al. [48] found *Leptotrichiaceae* and *Leptotrichia* (subtaxa of Fusobacteria) to be significantly lower in CeD than HCs. Iaffaldano et al. [48] also noted a lower abundance of Firmicutes, specifically *Lachnospiraceae* and *Veillonellaceae*, in CeD compared to HC, with Proteobacteria, *Neisseriaceae*, and *Neisseria* showing significant increases.

The focus on Proteobacteria, particularly *Neisseria flavescens*, is noteworthy because D’Argenio et al. [71] identified this species as the unique taxon representative of the MAM fingerprint in CeD, leading to differences at the own genus and family levels as well. Despite the broad spectrum of taxa (e.g., *Gemellaceae*, *Micrococcaceae*, *Prevotellaceae*, *Veillonellaceae*, and related subtaxa) exhibiting significantly reduced relative abundance in CeD, Panelli et al. [44] also reported that Proteobacteria, *Neisseriaceae*, and *Neisseria* were the only enriched taxa. Bodkhe et al. [72] also discussed various taxa that significantly differed between CeD and HCs, although their MAM fingerprint did not overlap with that presented by the aforementioned study [44]. It should be noted that both research groups based their investigations on comparisons against controls without a diagnosis of CeD but suffering from other pathologies, such as Hepatitis B Virus, functional dyspepsia, or gastroesophageal reflux, a condition that may have influenced the comparison and, consequently, the resulting significant findings.

A more recent study examined MAM according to a sampling of different parts of the duodenum, i.e., D1–D3 [73]. Authors observed a different fingerprint for each part with few taxa exhibiting common significance more than one time. However, it should be noticed how among these there was a decrease in *Moraxella* in both D1 and D2, a decrease in *Staphylococcus* in all the three sections profiled, a decrease in *Methylobacterium* in D2 and D3, an increase in *Prevotellaceae* (or related subtaxa) in D1 and D2, and an increase in *Neisseria* in D2 and D3 [73].

The study carried out by Wacklin et al. did not provide any details about differences in terms of taxonomy identification [74]; however, authors noticed a significantly different MAM composition between CeD and HC based on multivariate plotting according to unweighted Unifrac metrics.

As observed in studies on pediatric CeD, as well as profiling the MAM in adult CeD, three different studies did not report significant differences [52,75,76].

## 5. Is GFD Sufficient to Promote GM Eubiosis in CeD Patients?

Although CeD prevalence has been linked to the quantity and timing of gluten introduction into an infant’s diet [77,78], the international scientific community agrees that, after diagnosis, 20 ppm of gluten-derived antigens is the reasonable threshold to minimize the life-long risk of histological abnormalities in patients [79].

Foods and nutrition exert a critical role on human health, with a considerable attention devoted to understanding how nutrients and functional ingredients impact human physiology and responses. Clark and Mach [80] enlightened how changes in diet can be responsible for up to 57% of alterations in the GM, while host genes contribute to no more than 12% of these changes. Therefore, there has been increasing recognition of the interplay between GM and host health and diseases, which has led to a burgeoning interest in unraveling how foods regulate GM and consequently affect host homeostasis [81,82].

To promote GM homeostasis, individuals must adopt a healthy lifestyle consuming a diverse diet rich in fiber, fruits, and vegetables, limiting the intake of processed foods and added sugars, getting regular exercise, managing stress levels, avoiding unnecessary antibiotic use, and maintaining good hygiene practices [83,84]. Most of these healthy habits are absent in GFD, where gluten deprivation in cereal-based foods leads to a decrease in fibers and requires the addition of additives substituting the viscoelastic properties of the gluten network [85,86], till to the point that GFD can be flagged as “potentially unhealthy” [87].

Concerning dietary fiber, also known as microbiota-accessible carbohydrates (MACs), this class comprises various sugars linked through glycosidic bonds and can include chemical groups such as acetyl and sulfate. Fiber is pivotally involved in promoting health because it provides essential energy sources for GM influencing its composition and function [88]. Studies showed that low intake of MACs can negatively impact GM diversity and host health, while a high consumption was associated with multiple benefits, including increased levels of short-chain fatty acids (SCFAs) [89]. Specific fibers can improve glucose metabolism and modulate immune responses against pathogens [90,91]. Overall, dietary fiber plays a crucial role in maintaining gut health and preventing various metabolic disorders [92,93]. Most of the microbes pivotally involved in the saccharolytic metabolism were found reduced in multiple studies on MAM among those examined above suggesting that keystone taxa were lost due to the CeD onset. Among these, lactobacilli and bifidobacterial species are the most studied due to their significant involvement in fiber metabolism and synthesis of beneficial molecules, such as SCFAs [94]. However, these taxa are not alone because additional microbes co-participate in fiber degradation according to cross-feeding mechanisms, such as *Ruminococcaceae*, *Lachnospiraceae* [95], and *Prevotellaceae* [96]. Moreover, whatever the shift in GM metabolism, a reduced abundance of saccharolytic taxa led to an increase in proteolytic bacteria due to reduced acidification in the gut lumen [97].

Based on this depiction, De Palma et al. [98] evaluated the impact of GFD on the GM composition of ten HCs to avoid bias related to the pathophysiology featuring the CeD. They confirmed that GFD was featured by a lowering load of beneficial bacteria, such as *Bifidobacterium* and *Lactobacillus*, potentially due to the reduced availability of MACs serving as substrates for GM. Additionally, a reduction in *Faecalibacterium prausnitzii*, along with an increase in opportunistic pathogens such as *Enterobacteriaceae* and *E. coli* was observed confirming the bloom of proteolytic taxa [98]. Bacterial protein fermentation in the colon produces diverse metabolites, influenced by dietary protein content [99]. In fact, as found for metataxonomic differences, GFD has also demonstrated the capability to shift the metabolome and metabonome [100,101,102,103]. While proteins are crucial for microbial growth and beneficial metabolite production, recent advances in the field suggest that high-protein diets (HPDs), especially from animal sources, may have adverse health effects [99].

An additional key issue discussed in multiple ways by different authors is the risk that GFD can be characterized by increases in fats added to foods with the purpose of ameliorating their palatability [104,105,106]. Dietary fats provide a significant amount of calorie intake, impacting GM composition and function [107]. Omega-3 fatty acids have been found to reduce weight gain and inflammation by influencing GM, offering insights into mood and cognitive disorders [108]. Conversely, diets high in omega-6 fatty acids may increase inflammation, mediated by GM [109]. Saturated fats promote obesity and inflammation, while polyunsaturated fats, such as those in fish oil, offer protection [109]. High-fat diets can alter fecal bile acid profiles, potentially increasing the risk of enteric disease and promoting liver cancer through microbial metabolism [110,111].

## 6. Challenges and Future Directions in CeD Therapy

Based on the evidence discussed above, it is possible to speculate how in CeD there were two sides of the same coin leading to dysbiosis. On the one hand, in GCD, there is gluten that supports the growth of those proteolytic microorganisms involved in hydrolyzing immuno-cito-toxic peptides and epitopes and reducing the abundance of health-promoting bacteria and keystone-taxa in GM as the result of a generalized inflammatory status inducing dysbiosis [52,112]. On the other hand, in GFD, the lower dietary intake of fructans and arabinoxylans, i.e., fibers naturally occurring in wheat, represents a significant limitation for those microbes that mainly metabolize saccharides [113,114].

Hence, considering that GFD is still the unique therapy available to treat CeD after diagnosis, a field of research is working with the aim to define and provide additional alternatives capable of supporting GM and, in turn, health in patients.

According to a significant body of literature, both probiotics and prebiotics can be used to help, restore, and support a healthy balance of GM by modification of taxonomy and metabolome [115,116], as well as to produce metabolites involved in reducing the GM colonization from virulent pathobionts [117]. In fact, various strategies targeting GM have emerged to alleviate gluten-related disorders [118]. These methods primarily involved oral administration of specific bacteria, such as lactobacilli and *Bifidobacterium* spp. [119], or protein/peptide hydrolases such as glutenases [120,121]. Since irritable bowel syndrome (IBS) was found as a collateral condition featuring CeD [122] and probiotics were widely studied to treat IBS [123], a recent study demonstrated improvement in IBS-like symptoms among CeD patients adhering to a GFD following the oral intake of a multispecies combination of lactobacilli and *Bifidobacterium* [119]. However, while this treatment showed promise in reshaping GM, concrete evidence regarding microbial survival and effective gluten degradation under GIT conditions remained vague.

The clinical translation of microbial-based preparations has been hindered by various factors, including their poor survival in the GIT, the limited selection of lactobacilli and *Bifidobacterium* strains, and their inadequate activity against gluten epitopes [124,125,126]. Alternatively, protein/peptide hydrolases sourced from plants and microbes, such as *Bacillus stearothermophilus*, *Bacillus thermoproteolyticus*, *Bacillus licheniformis*, *Streptomyces griseus*, and *Aspergillus niger*, have been explored [76,121,127,128]. Enzyme-based therapies are investigated as innovative approaches aimed at inactivating immunogenic gluten peptides by peptidase supplementation. Microorganisms, both bacterial and fungal, serve as natural sources of peptidases, contributing to peptide digestion. However, the complete degradation of both gluten and its immunogenic derivatives by any single peptidase is challenging, and incomplete gluten hydrolysis may even worsen epitope accumulation rather than reduce it [128]. Therefore, formulating a combination of peptidases is crucial to developing effective treatments for preventing adverse reactions against accidentally ingested small amounts of gluten [76,115,120]. However, the efficacy of these formulations lacks substantial evidence, and their successful clinical application is limited by their susceptibility to GIT conditions. In line with these considerations, although the effectiveness under GIT conditions must be thoroughly validated to ensure their clinical utility [129], the scientific consensus favors novel microbial candidates, including spore-forming species, combined with traditional lactobacilli and *Bifidobacterium* strains, alongside proteolytic enzymes [115,130].

Since calcium, magnesium, iron, vitamin D, and fiber–in particular the soluble fraction–appeared to be limited in the GFD [131,132], additional studies in this field were based on specific supplementations, mainly testing various prebiotics [133,134,135,136,137] and suggesting a possible area of interventions based on promising results obtained.

An additional key issue featuring CeD is the high oxidative stress status observed in patients [138,139,140,141], which seemed not to be exclusively related to the diagnosis of CeD because it was found almost unchanged during the disease follow-ups despite the GFD [142]. In line with this, an antioxidant administration should be considered a valuable strategy to avoid or, at least, reduce cellular oxidation in CeD [143].

## 7. Challenges and Future Directions in MAM Sampling

As introduced, only a limited number of studies focused on MAM due to the need for ethical committee validation to proceed with biopsy collection for diseases where endoscopic methodologies are unnecessary for diagnosis or treatment. With this respect, a recent study opened new ways for sampling and analysis of MAM [144]. The authors compared lavage samples with mucosal brushing and biopsy samples collected from human subjects and found that lavage samples contained higher bacterial DNA levels and lower host DNA contamination compared to brush and biopsy samples. Although the bacterial composition in lavage samples was intermediate between that of feces and biopsies, bacteria abundant in biopsies were also enriched in lavage samples. These findings suggest that colonic lavage is suitable for analyzing MAM due to its minimally invasive nature and high bacterial DNA content, which could advance research on human MAM, particularly in GIT disorders, including CeD.

## 8. Conclusions

The host-gut microbiota interaction is a complex and dynamic relationship crucial for human health and well-being.

To the best of our knowledge, this is the first review specifically unveiling the mucosa-associated microbiota (MAM) fingerprint in CeD, despite the study of MAM holding significant importance in understanding its role in various diseases. The distinct microbial niches of the MAM and luminal microbes indicate their differential impacts on host physiology and immune responses. While the study of MAM presents methodological challenges, advancements in techniques such as colonic lavage offer promising avenues for minimally invasive sampling and comprehensive profiling of mucosal microbiota. Changes in microbial diversity and composition, characterized by alterations in bacterial taxa abundance and functionality, contribute to mucosal inflammation and compromised barrier function in CeD. Therefore, understanding the bidirectional interactions between MAM and the host immune system, especially in the context of CeD, is crucial for elucidating disease pathogenesis and identifying potential therapeutic targets.

In line with these considerations, future research efforts aimed at elucidating the complex interplay between MAM, host immunity, and environmental factors will facilitate the development of personalized strategies for managing CeD and other gastrointestinal disorders.

## Figures and Tables

**Table 1:** Significant differences (increase, black ↑, or decrease, red ↓) in MAM between pediatric celiac disease (CeD) patients under a gluten-containing diet (GCD) and non-celiac disease (HC) controls.

Reference	Journal	Year	Population	Country	Methods	MAM in CeD
Nadal et al. [60]	*J Med Microbiol*	2007	CeD = 30 HC = 8	Spain	16S rRNA-FISH	↑ Gram-negative bacteria ↑ *Bacteroides/Prevotella* ↑ *E. coli*
Schippa et al. [61]	*BMC Microbiology*	2010	CeD = 20 HC = 10	Italy	16S rRNA-TGGE	↑ *Bacteroides vulgatus* group ↑ *Clostridium coccoides* group ^$^ ↑ *E. coli*
Fernandez-Crehuet et al. [62]	*Anales De Pediatria*	2016	CeD = 11 HC = 6	Spain	16S rRNA-DGGE	↑ *Bacteroides* ↑ *Streptococcus* ↑ *E. coli* ↓ *Acinetobacter* ↓ *Bifidobacterium* ↓ *Lactobacillus*
Cheng et al. [63]	*Bmc Gastroenterology*	2013	CeD = 10 HC = 9	China	qRT-PCR	↑ *Prevotella melaninogenica* ↑ *Haemophilus* ssp. ↑ *Serratia* ssp.
Nistal et al. [57]	*Inflamm Bowel Dis*	2012	CeD = 8 HC = 5	Spain	16S rRNA gene seq	↑ *Neisseria (n.s.)* ^#^ ↑ *Prevotella (n.s.)* ↑ *Streptococcus (n.s.)*
Sánchez et al. [64]	*Appl Environ Microbiol*	2013	CeD = 32 HC = 8	Spain	16S rRNA gene seq	↑ *Firmicutes* ↑ *Proteobacteria*
Collado et al. [55]	*J Clin Pathol*	2009	CeD = 25 HC = 8	Spain	RT-PCR	↓ *Bifidobacterium* ↑ *Clostridium coccoides* group ^$^ ↑ *Clostridium leptum* group ^&^ ↑ *Bacteroides* ↑ *Staphylococcus* ↑ *Lactobacillus* ↑ *E. coli*
El Mouzan et al. [65]	*Gut Pathog*	2022	CeD = 20 HC = 19	Saudi Arabia	16S rRNA gene seq	↓ *Clostridiaceae* ↓ *Flavobacteriaceae* ↓ *Lactobacillaceae* ↑ *Micrococcaceae* ↓ *Clostridium* ↑ *Kocuria* ↑ *Lactobacillus* ↑ *Subdoligranulum* ↑ *Acinetobacter lwoffii* ↑ *Bifidobacterium angulatum* ↑ *Corynebacterium ihumii* ↑ *Corynebacterium tuberculostearicum* ↑ *Kocuria rhizophila* ↑ *Lactobacillus acidophilus* ↓ *Ralstonia pickettii* ↓ *Roseburia intestinalis*
Kalliomaki et al. [66]	*J Pediatr Gastr Nutr*	2012	CeD = 10 HC = 9	Finland	16S rRNA gene seq	No significant differences.
Ou et al. [67]	*Am J Gastroenterology*	2009	CeD = 45 HC =18	Sweden	16S rRNA gene seq	No significant differences.
de Meij et al. [68]	*Scand J Gastroenterol*	2013	CeD = 21 HC = 21	Netherlands	16S rRNA and 23S rRNA gene seq	No significant differences.

**Abbreviations:** CeD = Celiac disease; HC = healthy controls. ^$^ *Cl. coccoides* group (clostridial cluster XIVa, i.e., *Lachnospiraceae* family). ^&^ *Cl. leptum* group (clostridial cluster IV, i.e., *Ruminococcaceae* family). ^#^ n.s.: not significant (*p* > 0.05).

**Table 2:** Significant differences (increase, black ↑, or decrease, red ↓) in MAM between adult celiac disease (CeD) patients under a gluten-containing diet (GCD) and non-celiac disease (HC) controls.

Reference	Journal	Year	Population	Country	Methods	Significant Findings in CeD
Garcia-Mazcorro et al. [70]	*Nutrients*	2018	CeD = 6 HC = 12	Mexico	16S rRNA gene seq	↓ *Bacteroidetes* ↓ *Fusobacteria* (*p* = 0.052)
Iaffaldano et al. [48]	*Sci Rep*	2018	CeD = 14 HC = 20	Italy	16S rRNA gene seq	↓ *Bacteroidetes*; *Prevotellaceae*; *Prevotella* ↓ *Firmicutes*; *Lachnospiraceae Veillonellaceae* ↑ *Proteobacteria*; *Neisseriaceae*; *Neisseria* ↓ *Leptotrichiaceae*; *Leptotrichia*
D’Argenio et al. [71]	*Am J Gastroenterol*	2016	CeD = 20 HC = 15	Italy	16S rRNA gene seq	↑ *Neisseria flavescens* (at genus and family level, also)
Panelli et al. [44]	*J Clin Med*	2020	CeD = 52 HC * = 31	Italy	16S rRNA gene seq	↓ *Actinobacteria* ↓ *Bacteroidetes* ↑ *Proteobacteria* ↓ *Gemellaceae* ↓ *Micrococcaceae* ↑ *Neisseriaceae* ↓ *Prevotellaceae* ↓ *Veillonellaceae* ↓ *Parvimonas* spp. ↑ *Neisseria* spp. ↓ *Rothia* spp. ↓ *Streptococcus* spp. ↓ *Veillonella* spp.
Bodkhe et al. [72]	*Friont Microbiol*	2019	CeD = 23 HC * = 24	India	16S rRNA gene seq	↓ *Barnesiella* ↑ *Blautia* ↑ *Catenibacterium* ↓ *Eubacterium* ↑ *Helicobacter* ↓ *Intestinibacter* ↑ *Lactobacillus* ↑ *Megasphaera* ↑ *Methanomassiliicoccus* ↓ *Moraxella* ↑ *Prevotella* ↓ *Ruminococcus* ↓ *Turicibacter*
Constante et al. [73]	*Gastroenterology*	2022	CeD = 24 HC = 41	Italy	16S rRNA gene seq	(**D1**) duodenum section: ↓ *Acidovorax* ↓ *Dolosigranulum* ↑ *Escherichia/Shigella* ↓ *Moraxella* ↓ *Phenylobacterium* ↓ *Corynebacterium durum* ↑ *Dialister invisus* ↑ *E. coli* ↑ *Prevotella salivae* ↓ *Staphylococcus epidermidis* (**D2**) duodenum section: ↑ *Prevotellaceae* ↓ *Bacillus* ↓ *Bradyrhizobium* ↓ *Delftia* ↓ *Moraxella* ↓ *Methylobacterium* ↓ *Sellimonas* ↓ *Staphylococcus* ↑ *Collinsella aerofaciens* ↓ *Fusobacterium nucleatum* ↑ *Odoribacter splanchinus* ↓ *Veillonella parvula* ↑ *Neisseria sublava* ↑ *Prevotella salivae* (**D3**) duodenum section: ↑ *Acinetobacter* ↓ *Leuconostoc* ↓ *Methylobacterium* ↑ *Neisseria* ↓ *Phenylobacterium* ↑ *Peptostreptococcus* ↑ *Peptostreptococcus stomatis* ↓ *Staphylococcus epidermis*
Wacklin et al. [74]	*Inflamm Bowel Dis*	2013	CeD = 33 HC = 18	Finland	16S rRNA-DGGE and 16S rRNA gene seq	Differences were found between groups in terms of unweighted Unifrac metrics. However, a single taxon comparison was not performed.
Caminero et al. [52]	*Nat Commun*	2019	CeD = 12 HC * = 8	Canada	16S rRNA gene seq	Differences were not assessed by comparison between groups.
Nistal et al. [75]	*J Appl Microbiol*	2016	CeD = 9 HC = 9	Spain	16S rRNA gene seq	No significant differences.
Herran et al. [76]	*Res Microbiol*	2017	CeD = 5 HC = 7	Spain	16S rRNA-DGGE	No significant differences.

**Abbreviations:** CeD = Celiac disease; HC = Healthy controls. * HC = Controls without a diagnosis of CeD by suffering from other pathologies.

## Supplementary Materials

The following supporting information can be downloaded at: https://www.mdpi.com/article/10.3390/nu16111649/s1, Figure S1: Systematic review flow diagram detailing the database searches, the number of abstracts screened, and the full texts retrieved.

## Appendix A

The literature search was conducted using the pipeline (“celiac disease” OR “coeliac disease” OR “CeD” OR “CD”) AND (“biopsy” OR “mucosa adhering” OR “duodenal”) AND (“microbiota” OR “microbiome”) in “all fields” of three different databases PubMed (n. 179), Scopus (n. 303), and Web of Science (n. 50) for articles published until March 2024. Without introducing any a priori filter, a total of 532 research items were collected. After removing duplicates, book chapters, editorial letters, reviews, and all works that were not written in English, the number of studies was reduced to 277, as detailed in Supplementary Figure S1. These articles were manually checked by reading to focus specifically on literature that examined the impact of CeD on MAM in patients following gluten-containing dietary regimens. Therefore, a final number of 21 original articles filled the purpose of the present review, which were elaborated and discussed in Section 4.3.1 and Section 4.3.2.
