# Supplementary figures and images for: Celiac Disease: The Importance of Studying the Duodenal Mucosa-Associated Microbiota
Source: Nutrients. 2024 May 27;16(11):1649. doi: 10.3390/nu16111649 (PMC11174386; doi:10.3390/nu16111649)

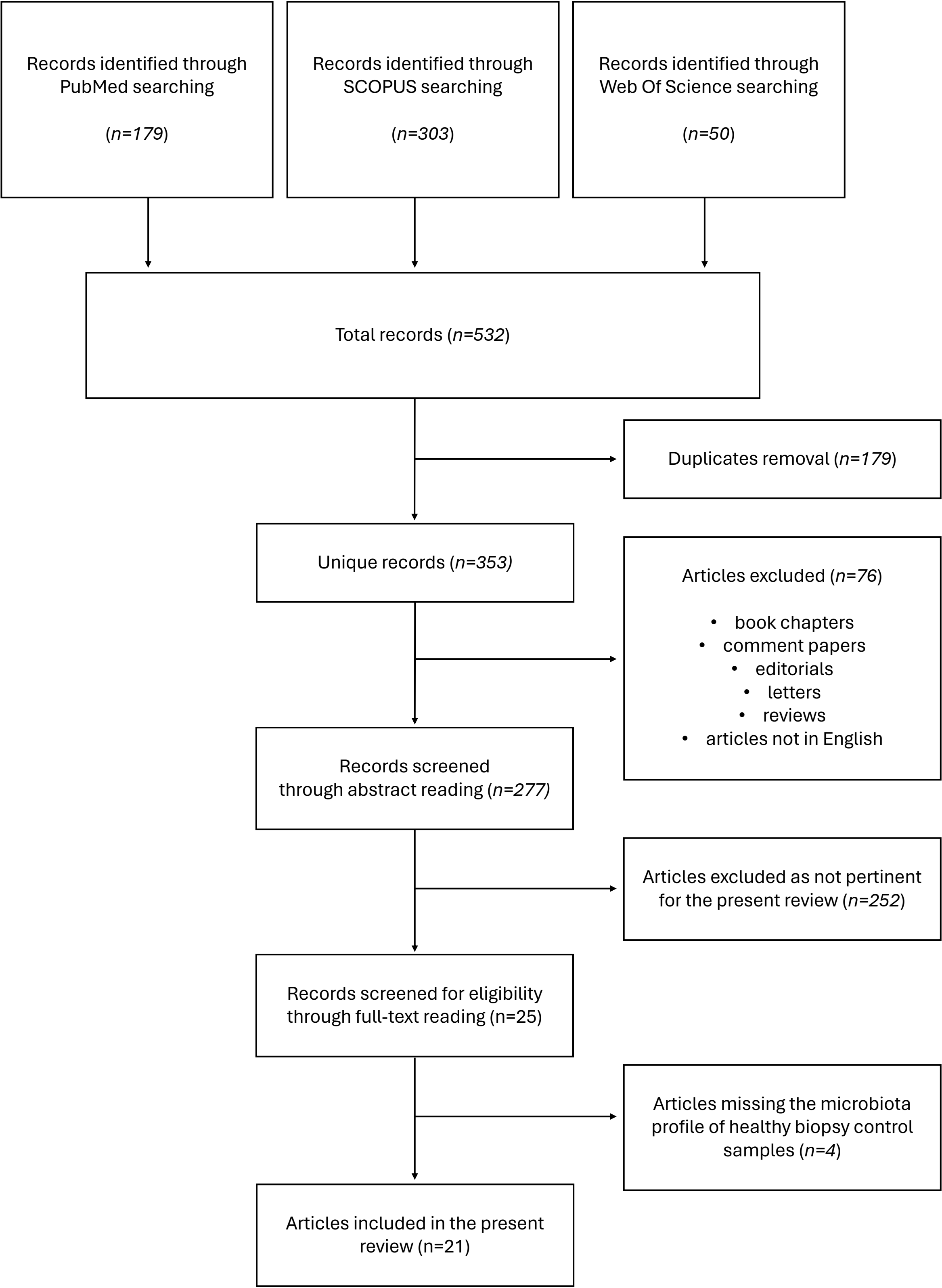

Supplement: Supplementary file 1 [file nutrients-16-01649-s001.zip › Supplementary Figure S1.pdf]
